# Three cases of laparoscopic total gastrectomy with intracorporeal esophagojejunostomy for gastric cancer in remnant stomach

**DOI:** 10.1186/1477-7819-12-342

**Published:** 2014-11-13

**Authors:** Yu Pan, Yi-Ping Mou, Ke Chen, Xiao-Wu Xu, Jia-Qin Cai, Di Wu, Yu-Cheng Zhou

**Affiliations:** Department of General Surgery, Sir Run Run Shaw Hospital, School of Medicine, Zhejiang University, 3 East Qingchun Road, Hangzhou, 310016 Zhejiang Province China

**Keywords:** Laparoscopy, Gastrectomy, Gastric remnant cancer, Intracorporeal anastomosis, Esophagojejunostomy

## Abstract

Gastric cancer in remnant stomach is a rare tumor but with poor prognosis. Compared with conventional open surgery, laparoscopic gastrectomy has potential benefits for these patients due to advantages resulting from its minimally invasive approach. Herein, we report on three patients with gastric cancer in remnant stomach who underwent laparoscopic total gastrectomy with intracorporeal esophagojejunostomy successfully. The operative time was 280, 250 and 225 minutes, the estimated blood loss was 100, 80 and 50 ml and the length of postoperative hospital stay was seven, eight and nine days respectively. Our experience has suggested that laparoscopic total gastrectomy with intracorporeal esophagojejunostomy can be a safe, feasible and promising option for patients with gastric cancer in remnant stomach.

## Background

Gastric cancer in remnant stomach is categorized as carcinoma arising from the remnant stomach after partial gastrectomy, regardless of the histology of the primary lesion (benign or malignant) or its risk of recurrence [1]. It has a poor prognosis and surgical resection remains the only effective modality of treatment. Most patients cannot withstand the huge blow caused by conventional open surgery because of their poor general condition. Therefore, laparoscopic surgery, well known for its minimally invasive advantages, is likely a preferable choice for these patients. However, the technical difficulty required for the procedure remains the major concern, especially for the safety of laparoscopic adhesiolysis and intracorporeal esophagojejunostomy. Our surgical term introduced the laparoscopic technique on the gastric cancer in remnant stomach successfully in our department based on our experience in the laparoscopic approach for diseases of digestive tract [2–5]. Herein, we report three cases of laparoscopic total gastrectomy with intracorporeal esophagojejunostomy (LTGIE) for gastric cancer in remnant stomach, with detailed operative procedures to evaluate its safety and feasibility, as well as to summarize surgical experience. This study protocol was prospectively approved by the ethics committee of Sir Run Run Shaw Hospital, School of Medicine, Zhejiang University and informed consent was signed by each patient prior to surgery.

## Case presentation

### Case 1

The patient was a 55-year-old male who underwent a distal gastrectomy with a Billroth II gastrojejunostomy for a peptic ulcer 30 years previously. The patient underwent a gastroscopy because of abdomen discomfort and a mass was found at the lesser curvature of the remnant stomach. A histological examination confirmed adenocarcinoma and a further examination did not reveal any distant metastasis.

**Figure 1:**
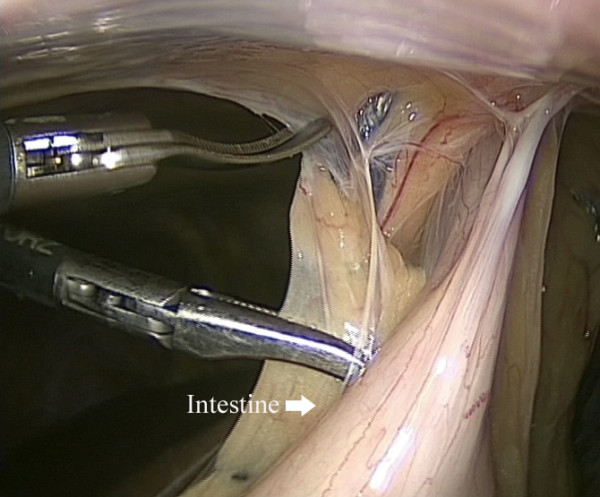
**Separation of adhesions between the bowels and abdominal wall.**

**Figure 2:**
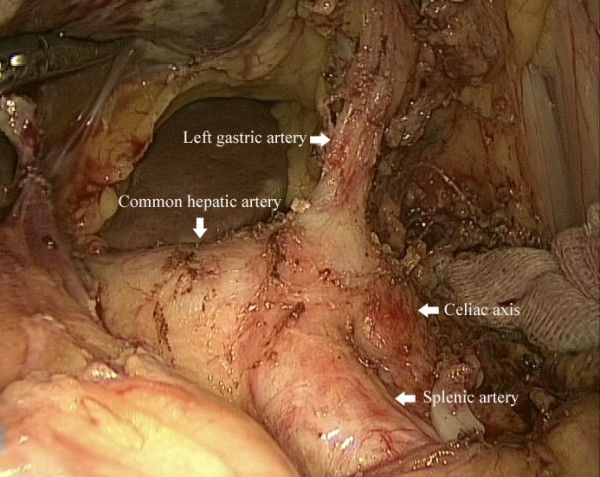
**Exposure of the common hepatic artery, splenic artery and the left gastric artery.**

**Figure 3:**
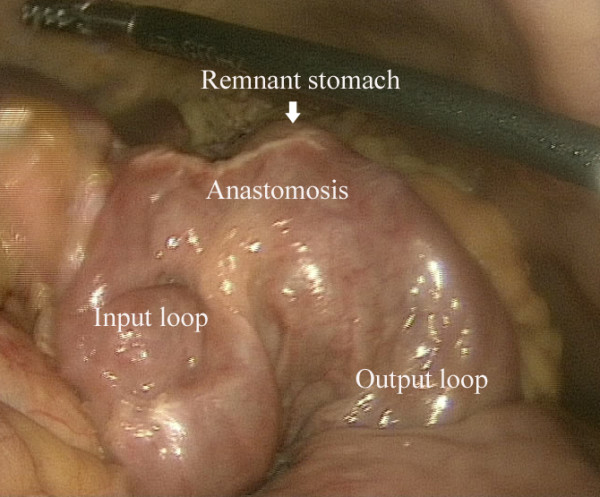
**Isolating the gastrointestinal anastomotic site.**

The patient’s position and the placement of trocars were similar to our previous studies [6]. The harmonic scalpel and scissors (Harmonic Ace scalpel, Ethicon Endo-Surgery, Inc, Cincinnati, OH) were used to separate the adhesions between the bowels, liver and abdominal wall (Figure 1). The remaining lesser omentum was dissected, exposing the common hepatic artery, splenic artery and the left gastric artery (Figure 2). The left gastric artery was dissected at the origin. After isolating the gastrointestinal anastomotic site (Figure 3), the input and output jejunal loops were lifted. With endoscopic linear staplers (Endocutter 60 staple; Ethicon Endo-Surgery, Inc, Cincinnati, OH), both input and output jejunal loops were transected at a point 5 cm distal to the anastomotic site. The remaining omentum was isolated to the splenic flexure. The short gastric vessels and the gastrosplenic ligament were dissected, mobilizing the stomach and exposing the esophagus.

**Figure 4:**
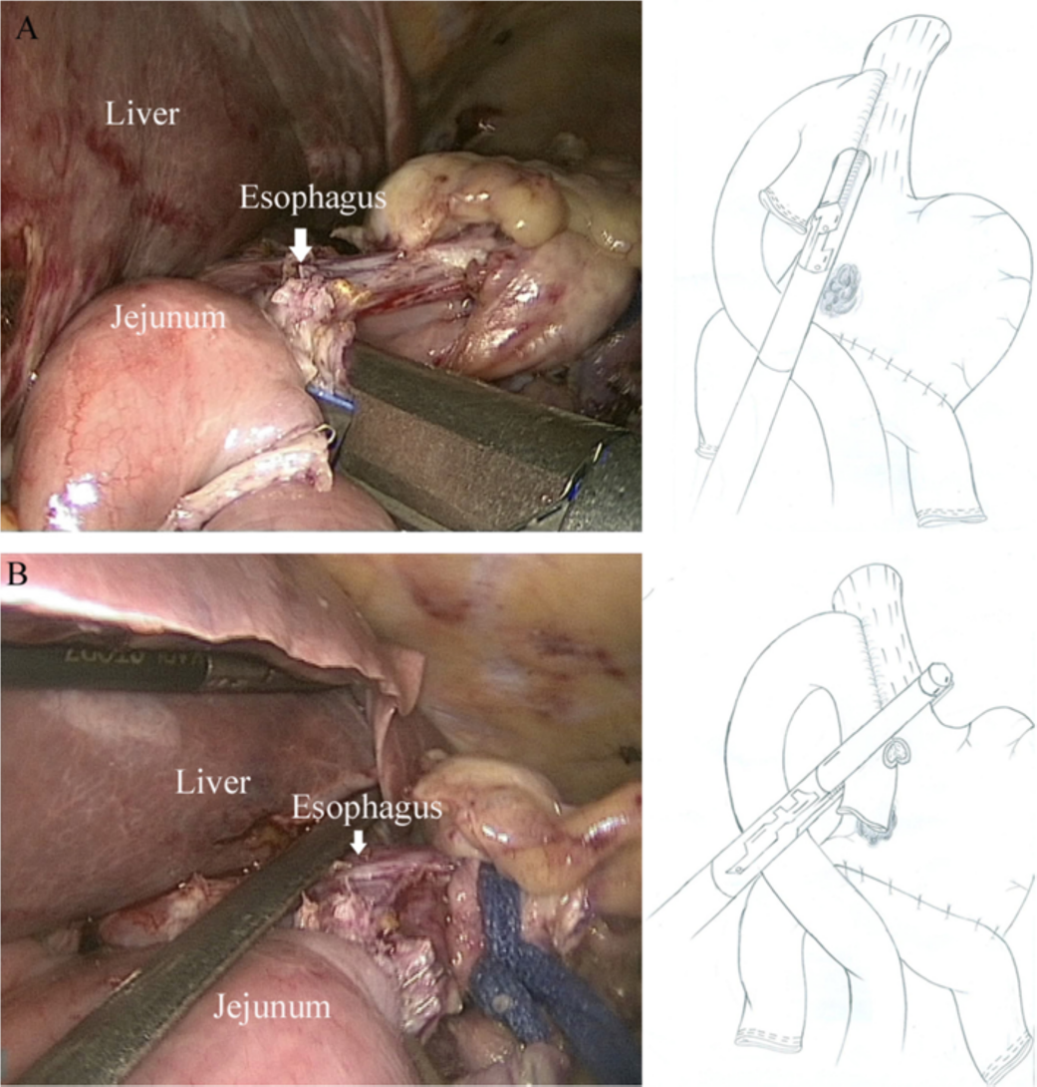
**Side-to-side intracorporeal esophagojejunostomy. A**. Each jaws of linear stapler is inserted into holes on the esophageal stump and the jejunum, then the linear stapler is fired. **B**. The common opening is closed using linear stapler.

An intracorporeal esophagojejunostomy was performed using endoscopic linear staplers. Two small holes were created; one on the anti-mesenteric side of the jejunum and the other on the esophageal stump. Each jaw of the endoscopic linear stapler was inserted into the holes. After stapling, a side-to-side esophagojejunostomy was constructed (Figure 4A). The common opening was closed and the esophagogastric junction was divided using endoscopic linear staplers (Figure 4B). The side-to-side enteroenterostomy to the Roux loop was made about 35 cm below the esophagojejunal anastomosis using an endoscopic linear stapler. The common opening was closed using the hand-suturing technique.

### Case 2

**Figure 5:**
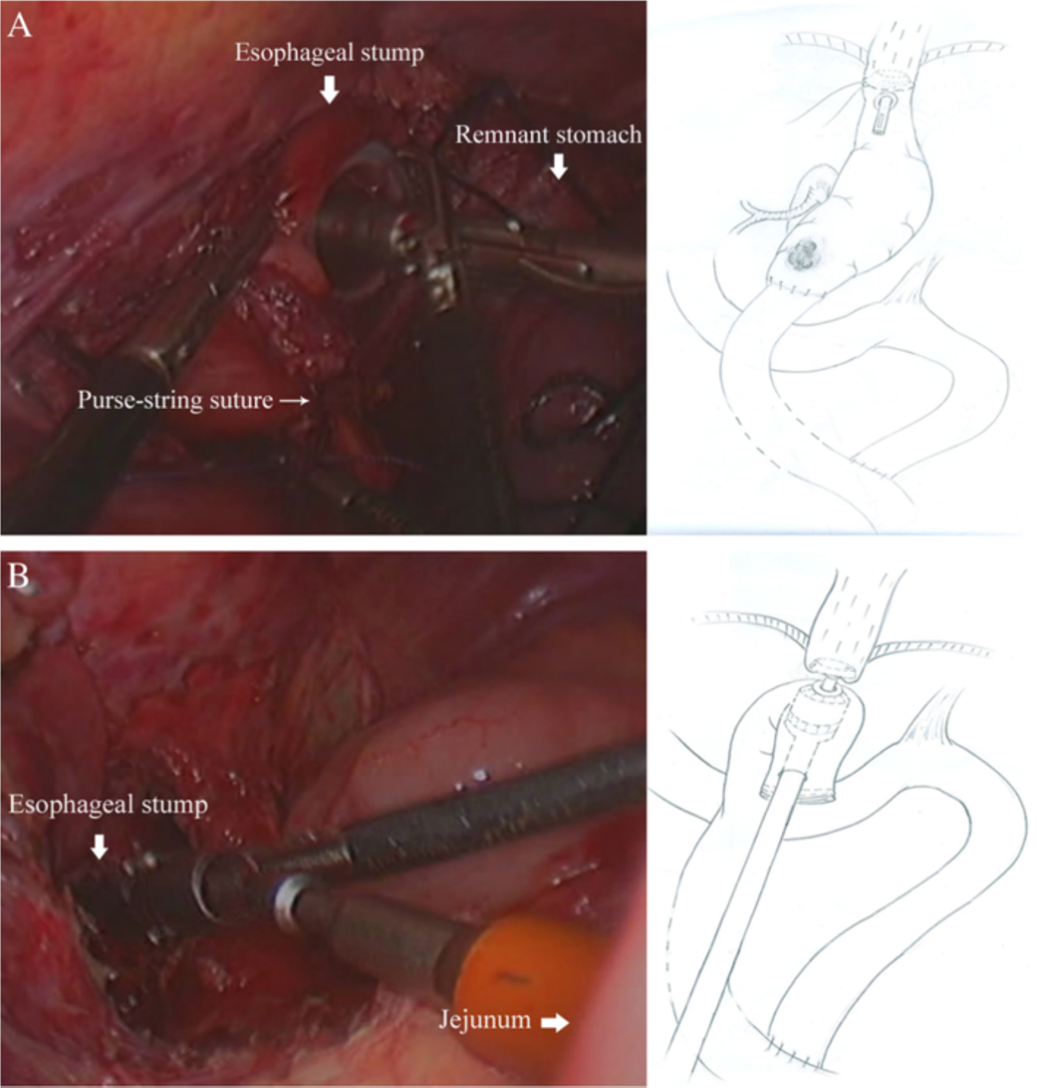
**End-to-side intracorporeal esophagojejunostomy. A**. The anvil is introduced into the esophageal stump through the hole, then the purse-string suture is tightened. **B**. The circular stapler is introduced into the jejunum through the jejunal stump, attached with the anvil and fired.

The patient was a 76-year-old male who underwent radical subtotal gastrectomy with a Roux-en-Y gastrojejunostomy for gastric cancer six years previously. The latest annual review of gastroscopy revealed a lesion in the remnant stomach and a histologically confirmed adenocarcinoma. Preoperative examinations did not show any distant metastasis.Dividing adhesion and mobilizing the stomach similar to case 1, the remnant stomach was lifted up; a purse-string suture was placed on the esophagus. A hole was made at the esophagogastric junction using a harmonic scalpel. The anvil (ECS 25, Ethicon Endo-Surgery, Inc, Cincinnati, OH) was introduced into the esophageal stump through the hole, then the purse-string suture was tightened (Figure 5A). The esophagogastric junction was divided and the remnant stomach was extracted. The circular stapler was introduced into the jejunum through the jejunal stump, attached with the anvil and fired (Figure 5B). The jejunal stump was closed with endoscopic linear staplers.

### Case 3

A 75-year-old male was admitted for repeated abdominal pain. He underwent a distal gastrectomy with a Billroth II gastrojejunostomy for a peptic ulcer 30 years previously. An adenocarcinoma near the anastomotic site of the stomach was found and a further examination did not find any distant metastasis. The patient underwent the operation as in case 1.

The three patients underwent entirely laparoscopic procedures without conversion. The operative time was 280, 250 and 225 minutes, the estimated blood loss was 100, 80 and 50 ml and the number of retrieved lymph nodes was 18, 10 and 22, respectively. Their postoperative course was uneventful; the three patients were discharged at postoperative day seven, eight and nine respectively. One patient is alive with no evidence of tumor recurrence. One patient (case 1) suffered tumor recurrence at 14 months after the operation and died at 17 months after the operation. One patient (case 2) died from Alzheimer’s 19 months after the operation, with no evidence of tumor recurrence. The clinical characteristics, surgical outcomes and long-term outcomes are shown in Table 1.

**Table 1:** **Clinical characteristics and outcomes of the three patients**

	Case 1	Case 2	Case 3
Age/sex	55/M	76/M	75/M
Previous disease	PU	GC	PU
Prior anastomosis	B-II	R-Y	B-II
Time since prior operation (years)	30	6	30
Operation time (minutes)	280	250	225
Intraoperative blood loss (mL)	100	80	50
Retrieved lymph nodes	18	10	22
First flatus (days)	2	3	5
Postoperative oral intake (days)	3	4	5
Postoperative hospital stay (days)	7	8	9
Follow-up period (months)	17	19	14

GC, gastric cancer; PU, peptic ulcer; R-Y, Roux-en-Y; B-II, Billroth II.

## Discussion

The incidence of gastric cancer in remnant stomach is about 3 to 5% [7, 8]. Previous reports show gastric cancer in remnant stomach may be associated with the lower acidic environment in the gastric stump, duodenogastric reflux, Helicobacter pylori infection and Billroth II reconstruction [9, 10]. Gastric cancer in remnant stomach has often been described as having a poor clinical outcome, with five-year survival rates of between 7 and 33% in previous studies [11, 12], and surgery is still the mainstay way to cure. Many patients with gastric cancer in remnant stomach are aged, with denutrition from prior gastrectomy. Thus, it is a great challenge for them to undergo conventional open surgery uneventfully and some patient-friendly procedures, such as laparoscopic procedures, are needed. Laparoscopic procedures for gastric cancer have gradually gained acceptance worldwide, and especially in some East Asian countries it has become the preferably choice due to advantages resulting from its minimally invasive approach [13, 14].

However, the technical complexity of the procedure caused by adhesion and the anatomical deterioration has compelled many surgeons to discontinue their trials. Severe intraperitoneal adhesion was previously considered as contraindicative for laparoscopic procedures. With the development of the laparoscopic devices and surgical skill, laparoscopic procedures nowadays are performed in the treatment of postoperative adhesive ileus and incisional hernia [15, 16]. Thus, it is possible to perform laparoscopic adhesiolysis safely on patients with gastric cancer in remnant stomach. Moreover, the laparoscopic magnified view allows adhesiolysis to be more meticulous than open surgery. We also believe that insertion of the first trocar in the open method can reduce risk of intestinal injury, and great care is needed to avoid injuring the colonic arteries and the colon when separating severe adhesion between mesocolon and jejunum.

To date, a few laparoscopic procedures for gastric cancer in remnant stomach have been reported and most were laparoscopic-assisted gastrectomy (LAG) [17–19]. Kwon *et al*. reported LAG are technically feasible approaches for the management of remnant gastric cancer for experienced surgeons [20]. However, LAG requires mini-laparotomy, which appears to spoil it’s minimally invasiveness advantages. Some surgeons have reported that laparoscopic gastrectomy with intracorporeal anastomosis has advantages over LAG, such as better cosmesis, less pain and less intraoperative blood loss [21, 22]. Additionally, LAG is not suitable for obese patients or those with a short esophageal stump [23]. Forceful tension and limited vision will cause tearing of the structure near the anastomosis, leading to a higher risk of fistula. Therefore, we chose to perform LTGIE for our patients. In our studies, the patients’ postoperative outcomes are consistent with previous studies [24], with a fast recovery and short hospital stay.

The technical difficulty of intracorporeal esophagojejunostomy is another critical obstacle for surgical safety. To overcome this problem, some modified techniques have been reported [25, 26]. These methods help to simplify the procedure of reconstruction and shorten the operation time. The most representative method is the side-to-side approach using an endoscopic linear staple. With this method, the anastomosis is not dependent on the size of the esophagus or the jejunum, but the endoscopic linear staple. A large anastomosis can be easily achieved, imposing less risk of anastomotic stricture. In our study two cases were successfully performed with this method with no anastomosis-related postoperative complications. Also, one patient had a Roux-en-Y gastrojejunostomy during a prior operation in our study. To preserve the Roux limb as long as possible, considering proper length of the Roux limb to protect the esophagus from entero-esophageal reflux, an end-to-side anastomosis seemed to be the most suitable approach. As a representative technique for intracorporeal end-to-side esophagojejunostomy, the OrVil™ (anvil) technique facilitates the conventional intracorporeal anastomosis and is time-saving. However, this technique may bring the risks of oral bacterium infection and injure the esophagus during insertion and it was unavailable in our institution. Thus, we performed the intracorporeal anastomosis with a conventional circular stapler rather than the OrVil™ technique.

## Conclusions

LTGIE can be a safe, feasible and promising option for patients suffering from gastric cancer in remnant stomach, with advantages of being a less invasive procedure and having a faster recovery time.

## Consent

Written informed consents were obtained from the patients for the publication of this case report and any accompanying images.

## Abbreviations

LTGIE: Laparoscopic total gastrectomy with intracorporeal esophagojejunostomy
LAG: Laparoscopic-assisted gastrectomy.
